# Differential Diagnosis Strategy between Lower Extremity Arterial Occlusive Disease and Lumbar Disc Herniation

**DOI:** 10.1155/2021/6653579

**Published:** 2021-04-05

**Authors:** Shaofeng Yang, Yijie Shao, Qi Yan, Cenhao Wu, Huilin Yang, Jun Zou

**Affiliations:** Department of Orthopaedic Surgery, The First Affiliated Hospital of Soochow University, Suzhou, Jiangsu 215006, China

## Abstract

Considering the increasingly incidence rate of lower extremity arterial occlusive disease and difficult to distinguish from lumbar disc herniation, it is very necessary to exclude lower extremity arterial occlusive disease resulting in lower limb symptoms from lumbar disc herniation. More importantly, who have a higher risk of combining with lower extremity arterial occlusive disease and misdiagnosed as lumbar disc herniation? Why those patients are easy to be misdiagnosed as lumbar disc herniation? It is worth analyzing and discussing. The risk factors including age, gender, the medical history of high blood pressure, diabetes, smoking and coronary, pulse pressure, lumbar disc herniation segment and type, ankle-brachial index, and straight leg raising test were observed. The Oswestry disability index and the Japanese Orthopedic Association score were collected preoperative, six months after posterior lumbar interbody fusion and six months after vascular interventional treatment to evaluate the symptoms relief and surgical efficacy. There was a statistically significant difference (*P* < 0.01) in pulse pressure, ankle-brachial index, central disc herniation, and straight leg raising test between two groups. There was a high risk to missed diagnosis of lower extremity arterial occlusive disease and misdiagnosed as lumbar disc herniation when patients are with a mild central lumbar disc herniation, higher pulse pressure, lower ankle-brachial index, and straight leg raising test negative. Therefore, sufficient history-taking and cautious physical examinations contributed to find risk factors and attach importance to such patients and, further, to exclude lower extremity arterial occlusive disease from lumbar disc herniation using lower extremity vascular ultrasound examination.

## 1. Introduction

Lumbar disc herniation (LDH) is displacement of disc material (annulus fibrosis or nucleus pulposus) beyond the intervertebral disc space [1], causing low back and/or leg pain, which typically presents with lower back pain that radiates down one leg, and is often accompanied by numbness or tingling in the foot [2]. Meanwhile, most of the population (more than 80%) will experience low back pain at some point in their lives [3]. For the reason of that LDH is one of the main causes of low back pain [4], low back pain with neurological symptom of lower extremity, especially in orthopaedics, is often subconscious considered to be caused by LDH [5]. However, those patients with a mild LDH only in radiographic images combined with other lower extremity vascular disease, such as lower extremity arterial occlusive disease (LEAOD), with some similar symptoms of pain, numbness, chill, paresthesia, and claudication in LDH patients [6–8], are to be misdiagnosed or missed diagnosed and further to delay treatment and increase economic burden. Considering the increasing incidence rate of LEAOD and difficult to distinguish from LDH, it is very necessary to exclude LEAOD resulting in lower limb symptoms from LDH. What is worse, LEAOD is not familiar and sensitive for orthopaedic surgeons relatively. In this case, for the patients with lower limb symptoms in orthopaedic department, who have a higher risk of combining with LEAOD and misdiagnosed as LDH? Why those patients are easy to be misdiagnosed as LDH? Therefore, our research is aimed at analyzing potential risk factors including age, gender, the medical history of high blood pressure (HBP), diabetes, smoking, and coronary, pulse pressure (PP), LDH segment and type, ankle-brachial index (ABI), straight leg raising test (SLRT), and comparison analysis using a retrospective clinical study.

## 2. Methods

The clinical study was approved by the Ethics Committee of the First Affiliated Hospital of Soochow University, and written informed consents were obtained from all participants. Totally 126 patients who had LDH with lower extremity symptoms and symptoms relieved significantly after lumbar surgery of posterior lumbar interbody fusion (PLIF) by a skilled surgeon in our department between January 2012 and December 2014 were selected and defined as group A. In this period, totally 22 subjects defined as group B had LDH with lower extremity symptoms as well but no symptom relief after PLIF by the same surgeon and finally diagnosed as LEAOD and symptoms recovered via vascular treatment. The Oswestry disability index (ODI) [9] and the Japanese Orthopedic Association (JOA) [10] score were collected preoperative, six months after PLIF and six months after vascular interventional treatment by a questionnaire and used to evaluate the symptoms relief and surgical efficacy between group A and group B. The symptom alleviation rate was defined as the improvement rate (IR) of JOA score six months postoperative compared to preoperative level. IR = [(postoperative JOA − preoperative JOA)/(29–preoperative JOA)] × 100%. IR > 60% meant significant effect; IR < 25% indicated no effect.

All subjects were conformed criteria as follows: (1) chief compliant of lower extremity symptoms (pain, numbness, chill, paresthesia, and claudication) and with or without back pain; (2) LDH was found on T2-weighted magnetic resonance imaging (MRI); (3) herniated disc was removed completely after PLIF based on the results of MRI postoperative within one week; (4) IR < 25% six months after PLIF treatment was enrolled in group B; (5) LEAOD was diagnosed clearly using digital subtraction arteriography (DSA) or computed tomography angiography (CTA); (6) follow-up for more than one year and with complete follow-up data. Exclusion criteria were (1) only with back pain; (2) LDH with poliomyelitis, paralysis inborn.

Gender, age, HBP, diabetes, smoking, coronary, PP, LDH segment and type, ABI, and SLRT in group A and group B were collected, respectively. HBP, diabetes, and coronary were diagnosed previously in cardiovascular department. Patients with a five-year smoking history were registered. PP was measured as the average range between systolic and diastolic blood pressure and repeated three times. LDH segment and type were diagnosed blindly by three experienced orthopaedic surgeons on MRI preoperative in accordance with the location of disc herniation. According to Premanath's method [11], ABI was calculated as the ratio of systolic blood pressure of ankle and brachial artery.

Data were performed with SPSS17.0 statistical software. Measurement data (showed as mean ± SD) was analyzed by independent-samples *T* test. Chi-square test was applied in enumeration data. *P* < 0.05 indicated the difference was statistically significant between both groups.

## 3. Results

Totally 148 enrolled participants were included in this study. During an average follow-up of 14.27 ± 2.94 months, LEAOD occurred in 22 patients (account for 14.86%). The characteristics of all patients in group A and group B were summarized in Table 1. Clinical efficacy index of JOA, IR, and ODI preoperative and 6 months after PLIF were collected in Table 2. The index preoperative and 6 months after vascular interventional treatment in group B is shown in Table 3. Comparison of the general characteristics showed that there were no statistical significance in gender, age, HBP, diabetes, smoking, coronary, and LDH segment. However, the mean PP in group A was 49.93 ± 4.20 mmHg, which was notably lower than group B (*P* < 0.01). The ABI in group A was greater than that in group B (0.783 ± 0.11 vs. 0.601 ± 0.15). LDH type in both groups had a significantly difference (*P* < 0.01), especially in terms of central disc herniation. 26 cases (account for 20.63%) in group A suffered central disc herniation and in 14 of the 22 cases (63.64%) occurred in group B. Meanwhile, SLRT positive occurred in 103 (81.75%) cases in group A and had a significantly difference compared to group B (22.73%). These results above indicated that participants with LDH who had a higher PP, lower ABI, central disc herniation, and SLRT negative had an increased risk of misdiagnosis and missed diagnosis of LEAOD.

**Figure 1:**
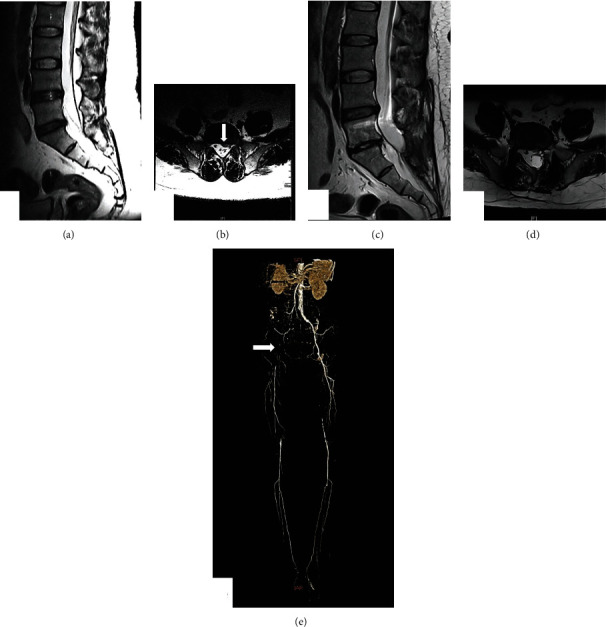
Radiographic data of the typical case. A 51-year-old male patient was hospitalized because of lower right limb pain and paresthesia. A higher PP (69 mmHg), lower ABI (0.62), and SLRT negative were found from physical examination. T2-weighted MRI sequence preoperative showed central LDH in L5-S1 (a, arrow in b). Herniated disc was removed completely after PLIF based on the results of MRI one week postoperative (c, d). Then, LEAOD was diagnosed clearly using CTA (e, arrow). MRI: magnetic resonance imaging; LDH: lumbar disc herniation; PLIF: posterior lumbar interbody fusion; LEAOD: lower extremity arterial occlusive disease; CTA: computed tomography angiography.

The clinical efficacy index of JOA and ODI six months after PLIF operation in group A had a significantly improvement, which had no notably difference in group B (Table 2). Meanwhile, IR in group A was as well significantly higher in group A than that in group B (63.44 ± 26.92 vs. 9.73 ± 6.56%). Patients in group B had a significantly symptoms relief (*P* < 0.01), and IR had reached an average of 52.99%, when receiving vascular interventional treatment (Table 3). These results showed that patients with LDH in group B had no effect after PLIF treatment and had a significantly symptoms relieve after vascular interventional treatment. Typical case is shown in Figure 1.

## 4. Discussion

LDH was a common, frequently occurring disease and was the main cause of lumbocrural pain in orthopaedic [3–5]. As its high incidence, LDH was the preferred consideration when patients with back pain and lower extremity symptoms. What was worse, patients were diagnosed as “LDH” to explain their lower limbs symptoms when disc herniation was just found on MRI even though a mild herniation, which would lead to a misdiagnosis and missed diagnosis of other real diseases causing similar lower limbs symptoms. With the enhancement of living standard and the change of dietetic habit, LEAOD was increasingly common, which had higher similar symptoms to LDH and thus difficult to distinguish from LDH. Therefore, it is necessary to analyze such patients and found out potential risk factors resulting in missed diagnosis and misdiagnosis. In this retrospective study, higher PP, lower ABI, central disc herniation, and SLRT negative had an increased risk of misdiagnosis and missed diagnosis of LEAOD.

Firstly, for the patients with LEAOD, systolic blood pressure had a significant increase, and there was no notably rise in diastolic blood pressure, which resulted in a significantly increase in PP [12]. In a previous study, Safar et al. reported that the increase of PP was significant than the average blood pressure raise [13, 14], which was conformed with the result of current study. There was no obvious difference in HBP but dramatically difference in PP between group A and group B. Secondly, lower ABI and SLRT negative were more common in LEAOD. ABI < 0.7 indicated vascular stenosis, which contributed to remind LEAOD exist [11]. SLRT positive occurred obviously in LDH patients, especially in L4-5 and L5-S1 disc herniation and usually could not found in LEAOD patients. Therefore, SLRT negative should be considered as a risk factor accompanied with LEAOD in LDH patients. Meanwhile, for the reason of that not all SLRT in patients with LDH were positive, it was needed to integrate other risk factors. Central disc herniation was a relative high incidence in LDH, less than lateral disc herniation, and could cause lower limbs symptoms or not, which lead to difficult to verify that whether those symptoms associated with central disc herniation. Lower symptoms were often considered firstly to be caused by LDH when patients had a central disc herniation, even though it was proved of the LEAOD influence. In this retrospective study, the PP in group B was much higher than that in group A, and their distinction was also statistically significant (*P* < 0.01). Patients in group B had a significantly lower ABI than that in group A. Meanwhile, the incidence of central disc herniation and SLRT negative in group B was much higher than that in group A. The authors concluded that it was a high risk to missed diagnosis of LEAOD and be misdiagnosed as LDH when patients with a mild central LDH, higher PP, lower ABI, and SLRT negative.

For the reasons of misdiagnosis as LDH, two factors of objective and subjective contributed to reveal its possible explanations. On one hand, the very similar symptom described above in LDH and LEAOD was indeed consisted of an important objective factor [6, 7]. In theory, there existed a situation that lower limbs caused by LDH combined with LEAOD, which would dramatically increase differentiating degree when continued to attempt to explain in one disease as usual. On the other hand, LEAOD was relatively strange to orthopaedic surgeons and thus difficult to consider even though a typical case. It should be stressed that the preconceived notion, especially with a mild disc herniation in orthopaedic that lumbocrural pain aroused by lumbar problems in many cases, would prevent us from considering other systems diseases. Additionally, depending on those radiographic such as MRI excessively and unable to differentiate the concepts of disc herniation with symptoms and without symptoms resulted in missed diagnosis and misdiagnosis as well [15].

Actually, it was easy to avoid misdiagnose or missed diagnosis as long as LEAOD was taken into account for such patients. There were several alternative imaging examinations including DSA, CTA, and magnetic resonance angiography (MRA). In addition, sufficient history-taking and cautious physical examinations contributed to find risk factors and attach importance to such patients, and further to exclude LEAOD from LDH using imaging examinations. For example, the skin temperature and color of affected limbs, the range of paresthesia, and the pulsation of dorsal foot artery and posterior tibial artery in patients with LEAOD were often abnormal. On the contrary, LDH patients generally have no abnormal skin color, the area of paresthesia generally runs along the nerve, the range was smaller than that of LEAOD patients, and the arterial pulsation was generally palpable.

The study has several limitations. Although this study had analyzed some potential risk factors, it did contain a limited amount. Some risk factors, such as nerve reflex and claudication, were needed to further compare between two groups. In addition, the retrospective design led to an inherent bias, which, together with the relatively small number of cases, may have made the results prone to error.

## 5. Conclusions

There was a high risk to missed diagnosis of LEAOD and be misdiagnosed as LDH when patients with a mild central LDH, higher PP, lower ABI, and SLRT negative. Therefore, sufficient history-taking and cautious physical examinations contributed to find risk factors and attach importance to such patients and, further, to exclude LEAOD from LDH using lower extremity vascular ultrasound examination.

## Figures and Tables

**Table 1:** Comparison of baseline characteristics of patients between two groups.

Index	Group A	Group B	*P* value
Gender (female/male)	46/80	9/13	0.69
Age (years)	49.13 ± 7.39	53.27 ± 5.05	0.084
PP (mmHg)	49.93 ± 4.20	59.73 ± 7.44	<0.001^∗∗^
ABI	0.783 ± 0.11	0.601 ± 0.15	<0.001^∗∗^
HBP (Y/N)	56/70	12/10	0.38
Diabetes (Y/N)	22/104	7/15	0.12
Smoking (Y/N)	69/57	10/12	0.42
Coronary (Y/N)	18/108	6/16	0.13
LDH segment			
L3-4	10	2	
L4-5	68	14	0.62
L5-S1	48	6	
LDH type			
Central	26	14	<0.0001^∗∗^
Beside central	25	2	
Lateral	73	6	
Far lateral	2	0	
SLRT (P/N)	103/23	5/17	<0.0001^∗∗^

PP: pulse pressure; ABI: ankle-brachial index; HBP: high blood pressure; LDH: lumbar disc herniation; SLRT: straight leg raising test; Y/N: yes/no; P/N: positive/negative. Compared with the other groups, ^∗∗^*P* < 0.01.

**Table 2:** Clinical efficacy index at 6 months after PLIF operation between group A and group B (mean ± SD).

Index	Group A	Group B	*P* value
JOA score			
Pre-	10.93 ± 2.98	11.40 ± 2.64	0.654
Post-	23.0 ± 3.29^##^	13.13 ± 2.55	<0.001^∗∗^
IR (%)	63.44 ± 26.92	9.73 ± 6.56	<0.0001^∗∗^
ODI (%)			
Pre-	41.86 ± 8.60	37.73 ± 5.06	0.119
Post-	27.46 ± 6.56^##^	33.71 ± 5.85	0.010^∗^

PLIF: posterior lumbar interbody fusion; JOA: Japanese Orthopedic Association; IR: improvement rate; ODI: Oswestry disability index; Pre-: before PLIF operation; Post-: 6 months after PLIF surgery; compared with the other groups, ^∗^*P* < 0.05; ^∗∗^*P* < 0.01. Compared with preoperative level, ^##^*P* < 0.01.

**Table 3:** Clinical efficacy index at 6 months after vascular interventional treatment in group B (mean ± SD).

Index	JOA score	ODI score
Pre-	13.93 ± 2.52	23.66 ± 3.53
Post-	22.2 ± 2.93	16.53 ± 2.54
IR (%)	52.99 ± 24.59	—
*P* value	<0.0001^##^	<0.0001^∗∗^

JOA: Japanese Orthopedic Association; IR: improvement rate; ODI: Oswestry disability index; Pre-: before vascular interventional treatment; Post-: 6 months after vascular interventional treatment; compared with preoperative level, ^∗∗^*P* < 0.01.

## Data Availability

The data used to support the findings of this study are available from the corresponding author on request.
